# Angiogenic activity mediates bone repair from human pluripotent stem cell-derived osteogenic cells

**DOI:** 10.1038/srep22868

**Published:** 2016-03-16

**Authors:** Li Zou, Qingshan Chen, Zachary Quanbeck, Joan E. Bechtold, Dan S. Kaufman

**Affiliations:** 1 Department of Medicine, University of Minnesota, Minneapolis, MN 55455, USA; 2 Excelen Center for Bone & Joint Research and Education, Minneapolis, MN, 55415, USA

## Abstract

Human pluripotent stem cells provide a standardized resource for bone repair. However, criteria to determine which exogenous cells best heal orthopedic injuries remain poorly defined. We evaluated osteogenic progenitor cells derived from both human embryonic stem cells (hESCs) and induced pluripotent stem cells (hiPSCs). Phenotypic and genotypic analyses demonstrated that these hESCs/hiPSCs are similar in their osteogenic differentiation efficiency and they generate osteogenic cells comparable to osteogenic cells derived from mesenchymal stromal cells (BM-MSCs). However, expression of angiogenic factors, such as vascular endothelial growth factor and basic fibroblast growth factor in these osteogenic progenitor cells are markedly different, suggesting distinct pro-angiogenic potential of these stem cell derivatives. Studies to repair a femur non-union fracture demonstrate only osteogenic progenitor cells with higher pro-angiogenic potential significantly enhance bone repair *in vivo*. Together, these studies highlight a key role of pro-angiogenic potential of transplanted osteogenic cells for effective cell-mediated bone repair.

Cell-based repair of non-union fractures and other bone defects is now commonly done using bone marrow-derived mesenchymal stromal cells (BM-MSCs)^1^,^2^. However, BM-MSCs must be isolated from various donors and are typically quite heterogeneous^3^,^4^. The application of BM-MSCs is limited by their finite proliferative potential and variation of donor-dependent repair potential, especially the cells isolated from aging adults^5^,^6^. Compared to BM-MSCs, human pluripotent stem cells (both human embryonic stem cells (hESCs) and induced pluripotent stem cells (hiPSCs)) provide a promising alternative cell source for bone repair and regeneration. *In vitro* and *in vivo* studies have confirmed that hESCs are potentially a good cell resource for studies of bone development and regeneration^7^,^8^,^9^. Recently, human iPSCs reprogrammed from different somatic cells have demonstrated the ability to generate osteoprogenitor cells with capability to form bone tissue *in vivo*^10^,^11^,^12^. The osteogenic potential of these cells derived from hESCs and hiPSCs enables them to be used as a novel resource for cell-based therapy of orthopedic injuries and other bone defects. Because of the genetic manipulation required to produce hiPSCs and the potential genetic and cellular instability of hiPSCs, tumorigenicity remains a potential hurdle for their *in vivo* application^13^,^14^,^15^,^16^. Although pluripotent stem cells and their differentiated derivatives show teratoma-forming propensity^17^,^18^, such risk is found to correlate with the residual undifferentiated pluripotent stem cells in the heterogeneous differentiated cell populations^19^,^20^,^21^. Therefore, it is crucial to fully differentiate pluripotent cells into the desired linage and carefully monitor the phenotypes of differentiated cells before *in vivo* application.

Bone vasculature also plays a vital role to mediate bone development and fracture repair^22^,^23^,^24^. In endochondral ossification, vascular invasion accelerates apoptosis of hypertrophic chondrocytes in the primary ossification center^25^,^26^. Inhibition of vascular invasion results in retarded bone growth with a large amount of hypertrophic chondrocytes in the growth plate and leads to poor fracture healing^23^,^26^,^27^. Because angiogenic factors regulate vascular invasion, various approaches have been employed to incorporate angiogenic factors, such as vascular endothelial growth factor (VEGF), basic fibroblast growth factor (bFGF) and bone morphogenic proteins (BMPs) into implanted cells or scaffolds to improve bone regeneration^28^,^29^,^30^. In addition to administration of exogenous growth factors, osteoblasts are known to produce VEGF to regulate bone remodeling by recruiting endothelial cells and osteoclasts^31^,^32^. Although angiogenic activity of MSCs and iPSCs has been suggested to contribute to their regenerative capability *in vivo*^33^,^34^,^35^,^36^, our understanding of the pro-angiogenic potential of osteogenic cells derived from pluripotent stem cells is relatively limited.

Here we examined the ability of osteogenic progenitor cells derived from hESCs and hiPSCs of different cell origins to promote bone repair. First, we evaluated osteogenic differentiation efficiency of two different hiPSC lines, derived from human peripheral blood mononuclear cells (PBiPSCs) and human umbilical cord blood CD34^+^ cells (UCBiPSCs), in parallel with hESCs. Osteogenic phenotypes and expression of key osteogenic genes, as well as production of angiogenic proteins, were compared with osteoprogenitor cells derived from hBM-MSCs *in vitro*. The ability of these hESC, hiPSC, and hBM-MSC-derived osteoprogenitor cells to repair bone *in vivo* was investigated using a rat femur non-union fracture model. The novel findings in these studies highlight that while the osteogenic cells from different sources have similar osteogenic phenotypes and characteristics *in vitro*, these cells markedly differ in their ability to promote vascular development *in vivo*. This pro-angiogenic activity of hESC/hiPSC-derived osteogenic progenitor cells plays a critical role in their ability to mediate effective *in vivo* repair.

## Results

### hESC and hiPSC-derived cells are similar in their osteogenic differentiation efficiency

**Figure 1:**
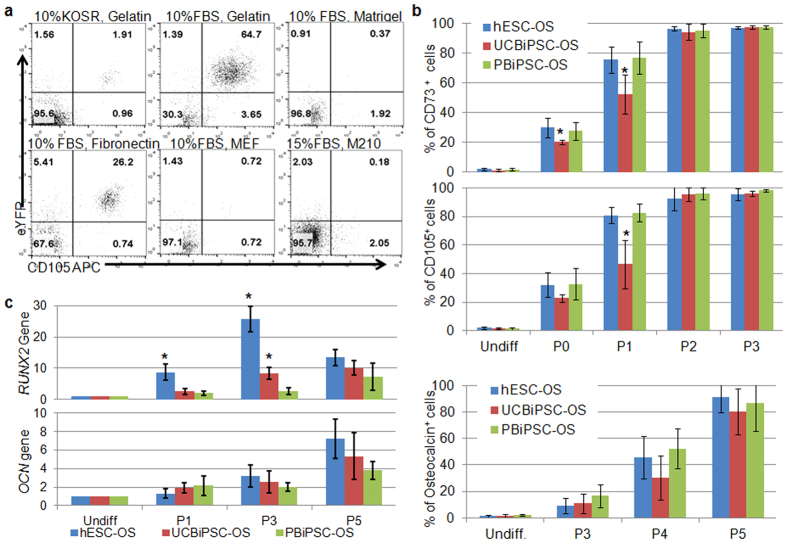
Differentiation of hESCs and hiPSCs in osteogenic conditions. (**a**) Flow cytometric analysis of hESCs differentiated in various conditions, with osteogenic supplements (dexamethasone, ascorbic acid, and β-glycerophosphate) in either 10% KOSR or 10% FBS, cultured on either gelatin, Matrigel, fibronectin, or by co-culture with MEFs or M210 stromal cells after 12 days of differentiation. A representative result is shown here from 3 independent experiments. (**b**) Summary of flow cytometric analysis of cell surface markers, CD73 and CD105, and intracellular osteogenic marker, osteocalcin, in the differentiated hESCs, UCBiPSCs and PBiPSCs cultured in the osteogenic medium with 10% FBS on gelatin at different passages. n = 3, *p < 0.05, One-way ANOVA with Tukey post-hoc tests for each comparing at each passage. (**c**) qRT-PCR analysis of osteogenic genes, *RUNX2*, Osteocalcin (*OCN*) in the differentiated cells. The gene expression level was normalized by undifferentiated cells for each cell line, and data was summarized from 3 independent experiments, *p < 0.05, One-way ANOVA with Tukey post-hoc tests for each comparing at each passage.

Using a RUNX2-YFP reporter-integrated hESC line previously used to better characterize hESC-derived osteogenic cells^9^, we initially optimized the osteogenic differentiation conditions to demonstrate that culturing these cells with 10% FBS and osteogenic supplements (dexamethasone, ascorbic acid and glycerophosphate) on 0.1% gelatin facilitates hESCs to generate more YFP^+^(Runx2^+^)/CD105^+^ osteogenic progenitor cells compared to other culture conditions (Fig. 1a). We then used this culture condition to mediate osteogenic differentiation of UCBiPSCs and PBiPSCs, two iPSC lines previously characterized in our group^37^,^38^ (supplemental Fig. 1). As demonstrated in previous studies, flow cytometric analysis for typical MSC surface antigens showed parallel development of CD73^+^ cells and CD105^+^ cells in cultures that mediate differentiation and expansion of these osteogenic cells derived from hESCs and iPSCs (termed hESC-OS, UCBiPSC-OS and PBiPSC-OS cells). After passage 3, differentiated hESCs and the two hiPSC lines are more than 95% of CD73^+^ and CD105^+^ cells (Fig. 1b). To evaluate osteogenic-specific differentiation, we quantified osteocalcin-expressing cells since osteocalcin is a biomarker of osteoblastic cells. Flow cytometric data demonstrated increasing osteocalcin^+^ cells with no significant difference among three cell lines (Fig. 1b). Quantitative RT-PCR analysis of osteogenic genes, *RUNX2*, osteocalcin (*OCN*) and osteonectin (*SPARC*), in the undifferentiated and differentiated cells demonstrated time-dependent increase in expression of osteogenic genes in the differentiated cells after serial passaging (Fig. 1c and supplemental Fig. 2). While the expression level of *RUNX2* is higher in hESC-OS cells at p1 and p3 than in other two cell lines, and *RUNX2* expression is also higher in UCBiPSC-OS cells than in PBiPSC-OS at p3, there is no significant difference in gene expression level of *RUNX2*, *OCN* and *SPARC* between the differentiated cells at p5 (Fig. 1c and supplemental Fig. 2). Together, these data suggest hESCs, UCBiPSCs and PBiPSCs are able to differentiate into osteoprogenitor cells with similar efficiency. For these studies, we term these osteoprogenitor cells produced under these conditions hESC-OS, UCBiPC-OS and PBiPSC-OS cells.

### Osteogenic differentiated human pluripotent stem cells possess similar phenotypes with osteoprogenitor cells derived from hBM-MSCs

**Figure 2:**
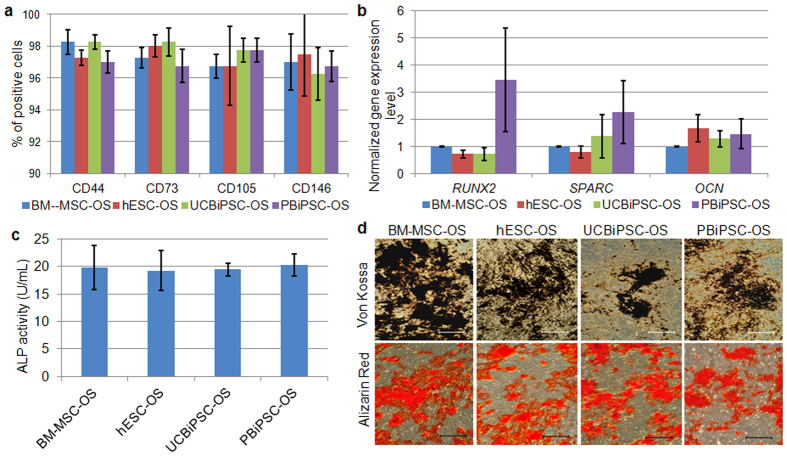
Osteogenic phenotypes of osteogenic differentiated hESCs and hiPSCs in comparison with BM-MSC derived osteoprogenitor cells. (**a**) Summary of flow cytometric analysis of CD44, CD73, CD105 and CD146 positive populations in osteogenic differentiated cells at passage 6. (**b**) qRT-PCR analysis of three osteogenic genes in the differentiated cells at passage 6. Gene expression level was normalized by the expression level in BM-MSC-OS group. (**c**) ALP activity assay of the osteogenic differentiated cells (2 × 10^5^) at passage 6 on day 3 after seeding. (**d**) Von Kossa staining and Alizarin Red staining of mineral deposition in the ECM around the differentiated cells (scale bar: 200 μm).

To evaluate the osteogenic potential of hESC-OS, UCBiPC-OS and PBiPSC-OS cells, we compared their appearance and phenotype with BM-MSC-OS cells (BM-MSCs further differentiated into osteogenic cells with FBS, dexamethasone, ascorbic acid and glycerophosphate). Morphologically, these osteogenic differentiated cells derived from hESCs and hiPSCs are indistinguishable with BM-MSC-OS cells (Supplemental Fig. 3). Flow cytometric analysis showed comparable homogenous cell populations in hESC-OS, UCBiPSC-OS, PBiPSC-OS and BM-MSC-OS cells, as all express MSC surface markers, CD44, CD73, CD105 and CD146, while lacking expression of hematoendothelial markers, CD31 and CD34 (Fig. 2a and Supplemental Fig. 4a). Gene expression analysis for *RUNX2*, *OCN* and *SPARC* also demonstrated similar expression level of these osteogenic genes in the differentiated cells (Fig. 2b). *In vitro* assays to assess osteogenic activity of the differentiated cells demonstrated similar alkaline phosphatase (ALP) activity and mineral deposition (Fig. 2c,d). To evaluate the osteogenic specificity of these differentiated cells, the osteogenic differentiated cells were cultured in adipogenic differentiation medium for 3 weeks, and no lipid droplets were detected (supplemental Fig. 4b). This is in contrast to abundant lipid droplets observed in undifferentiated BM-MSCs (not differentiated into OS cells) cultured in adipogenic medium as a positive control (supplemental Fig. 4b). Additionally, we also cultured the osteogenic differentiated cells (hESC-OS, UCBiPSC-OS, PBiPSC-OS and BM-MSC-OS) in chondrogenic differentiation medium, with BM-MSC (not differentiated into OS cells) as a positive control, using the protocol described in our previous study^8^. While BM-MSCs did form a cell aggregate and chondrogenic cells, none of the osteogenic differentiated populations formed a cell aggregate in chondrogenic conditions (data not shown). Based on these *in vitro* results, hESC-OS, UCBiPSC-OS and PBiPSC-OS cells possess osteogenic potential in a comparable level with BM-MSC-OS cells, but none of these OS populations form chondrocytes or adipocytes *in vitro*.

### Enhanced radiographic and biomechanical healing of rat femur non-union fracture repaired by osteoprogenitor cells derived from BM-MSCs and UCBiPSCs

**Figure 3:**
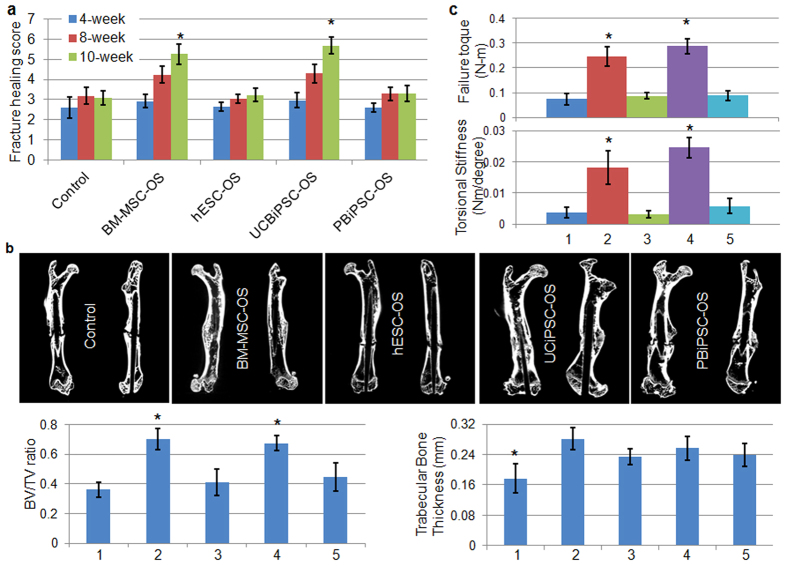
*In vivo* study of rat femur fracture repaired by osteoprogenitor cells derived from BM-MSC, hESCs and hiPSCs. (**a**) Quantitative evaluation of X-ray images of bone healing treated by different cells at different time points. n = 12 in each group at each time point, *indicates p < 0.05, One-way ANOVA with Tukey post-hoc tests. (**b**) Cross-sectional microCT images of healing femur treated by different cells at 10-weeks (upper panel) and bone morphometric analysis of bone formation at the fracture site in different groups (lower histogram); groups: 1-control, 2-BM-MSC-OS, 3-hESC-OS, 4-UCBiPSC-OS, 5-PBiPSC-OS. n = 5 for each group, *p < 0.05, One-way ANOVA with Tukey post-hoc tests. (**c**) Biomechanical testing of the healing bone tissue at 10-week after repair, groups: 1-control, 2-BM-MSC-OS, 3-hESC-OS, 4-UCBiPSC-OS, 5-PBiPSC-OS. n = 7 for each group, *p < 0.05, One-way ANOVA with Tukey post-hoc tests.

To test their capability to mediate bone repair *in vivo*, we used an established protocol to create a non-union fracture of rat femur^39^,^40^. In this fracture model, osteotomy followed by cauterization of periosteum made a more uniform fracture with stringent avascular microenvironment than by the alternative closed three-point bending fracture method^40^,^41^. Following fracture, we implanted an osteogenic progenitor cell-seeded gelatin sponge and observed fracture healing for 10 weeks. Notably, no teratomas or other abnormal growths were observed in any group when rat femurs were examined 10-weeks after surgery. X-ray examinations at 4-, 8-, and 10-weeks demonstrated progressive healing of the fractures repaired by BM-MSC-OS and UCBiPSC-OS cells, and less repair from the hESC-OS and PBiPSC-OS cells (supplemental Fig. 5a). Quantitative evaluation of femur fracture in terms of callus formation and fracture lines visibility in the X-ray images showed superior fracture healing in BM-MSC-OS and UCBiPSC-OS groups at 10-week, (scoring 5.26 ± 0.52 and 5.68 ± 0.43 versus 3.0 ± 0.35 in the control group), and little healing in hESC-OS and PBiPSC-OS treated groups, which were scored 3.22 ± 0.3 and 3.32 ± 0.39, respectively (Fig. 3a). Since such differential healing between groups are not fully demonstrated in the 3D reconstruction images of microCT (Supplemental Fig. 5b), we dissected the fracture healing by cross-section images, which clearly demonstrated that cortical bone continuity is successfully re-established in BM-MSC-OS and UCBiPSC-OS groups. Additionally, bone remodeling is noted in these BM-MSC-OS and UCBiPSC-OS groups, based on femur contour at the fracture site, alignment of fracture bone with adjacent bone tissue, and local mass of callus (Fig. 3b and supplemental Fig. 5a). In contrast, there remains a gap between the fracture ends in the no cell control group, hESC-OS and PBiPSC-OS groups, although bone callus is visible around the fracture sites in the PBiPSC-OS group (Fig. 3b and supplemental Fig. 5a).

Bone morphometric analysis of the healing tissue demonstrated significantly more bone formation in BM-MSC-OS and UCBiPSC-OS groups than the control group and the other two groups, indicated by BV/TV ratio, the bone volume fraction to show the mineralized bone in a given volume of interest, which are 0.36 ± 0.05, 0.70 ± 0.07, 0.40 ± 0.09, 0.67 ± 0.05, and 0.44 ± 0.09 for control group, BM-MSC-OS, hESC-OS, UCBiPSC-OS and PBiPSC-OS groups, respectively. However, the thickness of existing trabecular bone tissue is similar in the cell-engrafted groups, which are higher than the control group with no cells (Fig. 3b, histogram).

Biomechanical failure testing in torsion of the healing femur was consistent with the radiographic and micro-CT data. Failure torque and torsional stiffness confirmed that the mechanical properties and structural integrity of the healing femurs in BM-MSC-OS and UCBiPSC-OS groups are significantly better than other groups (Fig. 3c). Specifically, failure torque is 0.075 ± 0.023, 0.246 ± 0.04, 0.087 ± 0.012, 0.286 ± 0.031 and 0.088 ± 0.021, and torsional stiffness is 0.0036 ± 0.0016, 0.0181 ± 0.0054, 0.0032 ± 0.0011, 0.0245 ± 0.0032, and 0.0058 ± 0.0024 in the control group, BM-MSC-OS, hESC-OS, UCBiPSC-OS and PBiPSC-OS groups, respectively.

### BM-MSC-OS and UCBiPSC-OS cells promote bone healing through endogenous endochondral ossification

**Figure 4:**
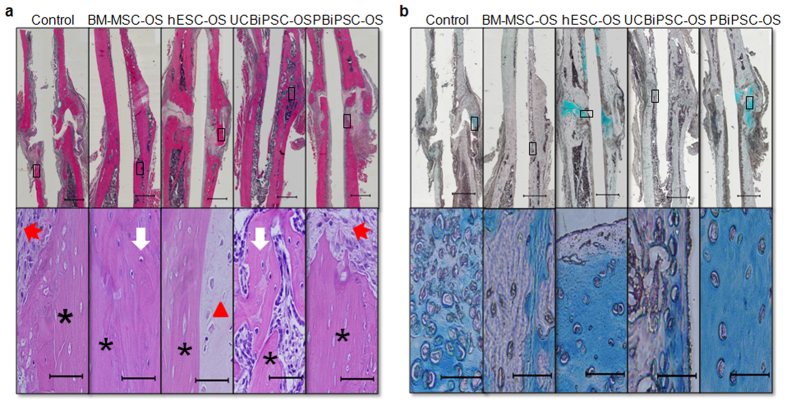
Histology of healing fracture repaired by different osteoprogenitor cells. (**a**) H&E staining of the healing femur treated by different osteoprogenitor cells at 10-weeks. Upper panel shows the scanned images of healing femur, scale bar: 1 mm; lower panel shows the healing tissue in the box under higher magnification, scale bar: 60 μm. Black asterisk indicates necrotic bone; white arrow indicates new bone tissue with osteoblasts and osteocytes; red triangle indicates cartilage tissue with chondrocytes; red arrow indicates connective tissue filling in the fracture ends. (**b**) Alcian Blue staining of glycosaminoglycan-rich cartilage tissue in healing femur at 10-week. Upper panel shows the scanned images of healing femur, scale bar: 1 mm; lower panel shows the healing tissue in the box under higher magnification, scale bar: 60 μm.

To better define the cell types and contribution of host cells and donor cells to mediate bone healing, histological evaluation and immunohistochemical staining were performed. H&E staining demonstrated stable bone union in BM-MSC-OS and UCBiPSC-OS groups, whereas there is a more fibrous union of the fracture in the control group, and hESC-OS and PBiPSC-OS groups (Fig. 4a upper panel). Higher magnification revealed development of neo-bone tissue with osteocyte-seeded laminar structures in BM-MSC-OS and UCBiPSC-OS groups, in contrast to the fibrous connective tissue or cartilage tissue in the control group, hESC-OS and PBiPSC-OS groups, which was located adjacent to the necrotic bone (Fig. 4a lower panel). Alcian blue staining to show cartilage tissue rich in glycosaminoglycans revealed a large amount of chondrocytes at the fracture sites in these three fibrous healing groups, especially hypertrophic chondrocytes locally concentrated at the fracture sites in hESC-OS and PBiPSC-OS groups, and a trace amount of cartilage tissue was also observed in femur fractures treated with BM-MSC-OS and UCBiPSC-OS cells (Fig. 4b). The finding of cartilage tissue in all groups demonstrated that bone repair of the non-union fracture is mediated by endochondral ossification mechanism. And presence of cartilage in the control group (no human cells engrafted) strongly suggested that the cartilage tissue is formed by the host cells.

**Figure 5:**
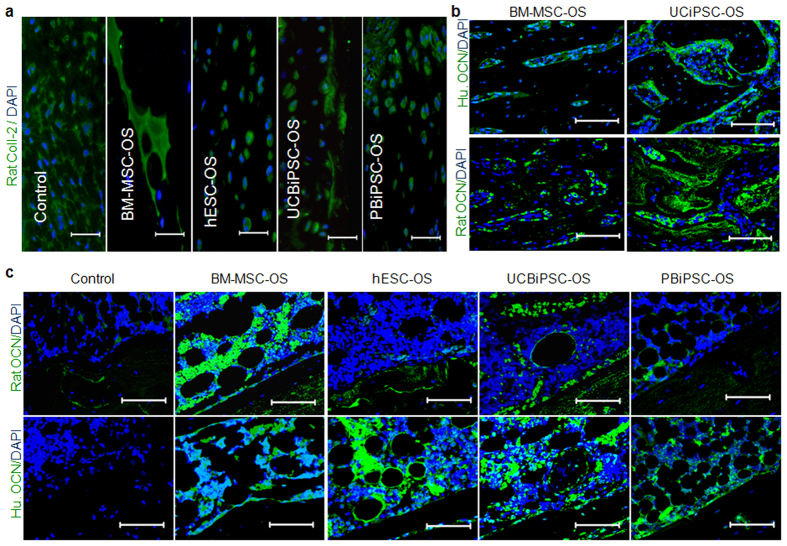
Immunohistochemical staining of cells in the implants and healing tissue. (**a**) Immunostaining of rat collagen-II (green) and DAPI (blue) in the healing tissue (scale bar: 25 μm). (**b**) Immunostaining of human or rat osteocalcin and DAPI in the healing bone repaired by BM-MSC-OS and UCBiPSC-OS cells at 10-week (scale bar: 80 μm). (**c**) Immunostaining of rat osteocalcin and DAPI (upper panel), human osteocalcin and DAPI (lower panel) in the implanted constructs at 10-week (scale bar: 80 μm).

To better define the origin of chondrocytes, we did immunostaining of the cartilage tissue using species-specific anti-rat collagen-II antibody, which confirmed the rat origin of cartilage tissue in the healing bone (Fig. 5a). To further identify the origin of the osteoprogenitor cells that contribute to bone repair in BM-MSC-OS and UCBiPSC-OS groups, we used osteocalcin antibodies which specifically recognize either human or rat species to stain bone tissue at the fracture sites in these two groups. The immunostaining results showed that human osteocalcin^+^ cells are mixed with rat osteocalcin^+^ cells, suggesting engrafted human osteoprogenitor cells and endogenous rat osteoprogenitor cells both contribute to fracture repair (Fig. 5b). Next, in order to determine if these host cells are recruited by human cells seeded in the implanted constructs, we evaluated the cells in the implanted constructs including those without human cells (negative control group). Immunostaining demonstrated that rat osteoprogenitor cells are recruited to line up on the surface of human cell-seeded constructs and even infiltrate into them, especially in BM-MSC-OS and UCBiPSC-OS groups that mediate better repair. However, there are very few rat osteocalcin^+^ cells in or around the implanted construct without human cells (Fig. 5c, upper panel). Immunostaining with human osteocalcin antibody also showed many osteocalcin^+^ cells in the implanted constructs (except the negative control without human cells) demonstrating the implanted human cells retain their osteogenic activity (Fig. 5c, lower panel). These immunostaining results clearly demonstrate that implanted human osteogenic cells recruit host cells to the fracture sites and the recruited rat cells contribute to fracture healing through endochondral ossification.

### Osteoprogenitor cells derived from human pluripotent stem cells have different pro-angiogenic potential

**Figure 6:**
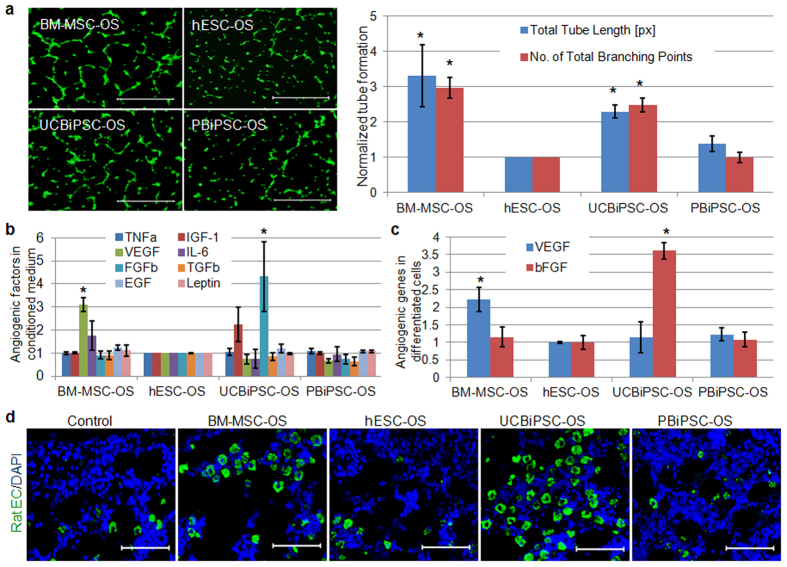
Pro-angiogenic potential of the osteogenic differentiated cells derived from BM-MSC, hESCs and hiPSCs. (**a**) Matrigel tube assay of HUVECs cultured in the conditioned medium from these osteogenic differentiated cells. Images acquired 4 hours after seeding HUVECs on growth factor reduced Matrigel (scale bar: 200 μm). The tube formation was quantitatively analyzed using Image J and normalized by the results in hESC-OS group, as summarized in the histogram. n = 4, *p < 0.05, One-way ANOVA with Tukey post-hoc tests, compared to the reading in hESC-OS group. (**b**) ELISA analysis of eight angiogenic factors in the conditioned medium from the osteogenic differentiated cells. Readings were normalized by the concentration in hESC-OS group. n = 3, *indicates p < 0.05 One-way ANOVA with Tukey post-hoc tests, compared to the reading in hESC-OS group. (**c**) qRT-PCR analysis of angiogenic genes, VEGF and bFGF, in the osteogenic differentiated cells at passage 6. Gene expression level was normalized by the result in hESC-OS group. n = 3, *indicates p < 0.05 One-way ANOVA with Tukey post-hoc tests, compared to the reading in hESC-OS group. (**d**) Immunostaining of rat endothelial cells and DAPI in the implanted constructs at 10-weeks (scale bar: 80 μm).

Since our *in vitro* results showed that these osteogenic cells derived from hESCs and hiPSCs demonstrated similar osteogenic activity as BM-MSC-OS cells, we next wanted to evaluate why implantation of BM-MSC-OS and UCBiPSC-OS cells results in better bone repair than the other cells *in vivo*. It is known that angiogenesis facilitates degradation of hypertrophic chondrocytes in endochondral ossification^25^,^26^. Previous studies with BM-MSCs demonstrate their angiogenic activity mediates tissue repair in ischemic diseases^33^,^34^,^42^,^43^. One intriguing recent report demonstrates that VEGF produced from MSCs can mediate healing of intestinal ulcers^36^. Therefore we tested the angiogenic potential of these hESC- and hiPSC-derived osteogenic cells. Flow cytometric data demonstrates that these osteoprogenitor cells are CD31^−^ (Supplemental Fig. 4a), illustrating these cells do not contain an endothelial cell component with angiogenic activity. To test the paracrine effect of these cells, we did functional test of the pro-angiogenic activity of the conditioned medium (CM) from these osteoprogenitor cells to mediate capillary-like tube formation from HUVECs. Interestingly, we found that HUVECs formed better capillary-like tubes when cultured in CM from BM-MSC-OS cells and UCBiPSC-OS cells, in contrast to less endothelial cell activity induced by CM from hESC-OS and PBiPSC-OS cells (Fig. 6a). Total tube lengths show a 3.3 ± 0.87, 2.29 ± 0.19, and 1.38 ± 0.21-fold increase in the groups treated by CM from BM-MSC-OS, UCBiPSC-OS and PBiPSC-OS cells in comparison with the cells treated by hESC-OS-derived CM. The number of total branching points demonstrated 3.0 ± 0.29, 2.4 ± 0.2, 0.98 ± 0.15-fold increase, respectively, in those groups, compared to the group of hESC-OS cells. To better define mediators of the angiogenic activity, ELISA analysis of eight soluble angiogenic factors in the CM from the different cell sources confirmed significantly more VEGF (3.1 ± 0.37-fold increase) in the medium from BM-MSC-OS cells and more bFGF (4.3 ± 1.5-fold increase) from UCBiPSC-OS cells, compared to those in hESC-OS cells (Fig. 6b). qRT-PCR analysis of angiogenic genes in the osteogenic cells showed that the expression level of VEGF demonstrated 2.2 ± 0.14, 1.14 ± 0.34, 1.22 ± 0.19-fold increase in BM-MSC-OS, UCBiPSC-OS and PBiPSC-OS cells compared to hESC-OS cells, and bFGF expression level demonstrated 1.15 ± 0.23, 3.5 ± 0.28, and 1.08 ± 0.24-fold increase in these cells, respectively, compared to hESC-OS cells (Fig. 6c).

Both ELISA and qRT-PCR results support the functional angiogenic data to demonstrate higher pro-angiogenic potential of BM-MSC-OS and UCBiPSC-OS cells than hESC-OS and PBiPSC-OS cells. To further validate the pro-angiogenic potential of these cells *in vivo*, we evaluated angiogenesis in the implanted constructs. Our immunostaining of rat endothelial cells revealed that more rat endothelial cells invade into the constructs in BM-MSC-OS and UCiPSC-OS groups than other groups (Fig. 6d), which is consistent with our *in vitro* results and provides a plausible mechanism of better bone repair with little cartilage tissue in these two groups.

Based on these *in vivo* studies, we concluded that host cells are recruited, at least partially, by the implanted human cells and contribute to endochondral ossification during fracture healing, and the pro-angiogenic potential of the implanted human cells plays an important role to determine the progress of endochondral ossification in bone repair, rather than repair being solely determined by the intrinsic osteogenic potential of the implanted cells.

## Discussion

Although hiPSCs share most attributes with hESCs, it remains unclear whether these two pluripotent stem cells have similar function compared with BM-MSCs. To address this question, previous studies have evaluated the capability of osteoprogenitor cells derived from these pluripotent stem cells to regenerate bone tissue but obtained variable results^10^,^11^,^12^,^44^. Bone vasculature is a pre-requisite for bone regeneration^22^,^24^; however, pro-angiogenic activities of the transplanted cells used for osteogenic repair are not typically investigated when evaluating their regenerative potential. Here, we induced osteogenic differentiation of three distinct pluripotent stem cell populations and compared the ability of the pluripotent stem cell-derived osteogenic cells to mediate bone regeneration *in vitro* and *in vivo*. We found that hESCs and hiPSCs demonstrated similar ability to generate homogenous osteoprogenitor cell populations with comparable osteogenic phenotypes to BM-MSC-derived osteoprogenitor cells. However, these osteoprogenitor cells were markedly different in angiogenic potential. *In vivo* studies using these osteoprogenitor cells to repair a rat femur non-union fracture showed that the osteoprogenitor cells derived from BM-MSCs and UCBiPSCs, which demonstrate higher pro-angiogenic activities, have superior capability to repair fracture by accelerating endogenous endochondral ossification with increased angiogenesis from host endothelial cells.

Although pro-angiogenic activity of hBM-MSCs is well documented and these multipotent cells have been employed in many clinic trials to treat patients with myocardial ischemia, stroke, peripheral vascular ischemia and other injuries, including orthopedic fractures^2^,^33^,^34^,^42^,^43^, our understanding of angiogenic potential of more lineage committed cells such as osteogenic progenitor cells is limited. Here, we highlight that angiogenic activity of osteoprogenitor cells derived from pluripotent stem cells plays a critical role to promote bone regeneration *in vivo*. Additionally, our results also demonstrate that 1) the engrafted human osteogenic cells may induce endogenous bone repair by recruiting cells from host tissue; 2) hiPSCs of different cell origins can differentiate into osteogenic lineage-specific cells with similar efficiency and phenotypes as hESCs; and 3) osteogenic activity of hiPSC- and hESC-derived osteoprogenitor cells can be indistinguishable based on *in vitro* characterization, but their ability to promote bone regeneration *in vivo* may still remarkably differ due to their differences in pro-angiogenic potential.

Bioactive molecules incorporated in an implanted biomaterial scaffold or secreted from transplanted cells may recruit adjacent host progenitor cells for endogenous tissue regeneration *in situ*^45^,^46^,^47^. Specifically in bone repair, these progenitor cells derived from MSCs typically regenerate bone through endochondral bone formation if the fracture is not rigidly fixed, such as what occurs with intramedullary fixation^1^,^48^,^49^. Usually, inflammatory factors released from the injured tissue or cytokines from the implanted cells may recruit host cells for local tissue repair^50^. In our *in vitro* study, we did detect growth factors secreted by the osteogenic progenitor cells, including TNF-α, IL-6, VEGF, TGF-β, bFGF, leptin, IGF-1, and EGF (Fig. 6b). When engrafted, these live cells are able to recruit host cells by a paracrine effect, which is confirmed by immunostaining results of rat osteocalcin positive cells in the implanted human cell-seeded constructs, but not in the no-cell seeded construct. Furthermore, our alcian blue staining and rat-collagen II specific immunostaining confirmed rat origin of the cartilage tissue in the healing bone, and osteocalcin immunohistochemical staining also showed mixed human and rat osteocalcin positive cells in the healing bone tissue repaired by BM-MSC-OS and UCBiPSC-OS cells (Fig. 5b). Therefore, these studies illustrate the recruited endogenous cells by implanted human cells participate in bone formation through endochondral mechanism to repair femur fracture in this study.

Skeletogenesis occurs in close proximity to vascular ingrowth that provides cytokines, chemokines, growth factors, and hormones required for bone formation^22^,^23^,^24^. It is known that disruption of the vasculature can lead to osteonecrosis and delayed fracture healing or non-union, and combining angiogenic and osteogenic factors may increase bone formation *in vivo* compared to either factor alone^27^,^51^,^52^,^53^, again demonstrating the tight association of bone regeneration with angiogenesis. In this study, we created a stringent avascular microenvironment by cauterizing the periosteum 2 mm proximally and distally to the fracture ends after osteotomy^39^,^40^. As expected, blockage of the blood supply led to non-union of the fracture, as seen in the no-cell control group. Our *in vitro* results consistently demonstrated the superior pro-angiogenic potential of BM-MSC-OS and UCBiPSC-OS cells, which was further verified by *in vivo* immunostaining of rat endothelial cells in the implanted constructs. Although these two cell lines express different angiogenic growth factors, higher VEGF in BM-MSC-OS cells and more bFGF in UCBiPSC-OS cells, these two growth factors are well-documented potent angiogenic factors to regulate bone development and repair^54^,^55^,^56^. Therefore, it is not surprising that engraftment of these two cells significantly enhances bone repair in the stringent avascular microenvironment after osteotomy of rat femur. Although angiogenesis is an important mediator of cell-based therapies, relatively little attention has been paid to the pro-angiogenic activity when evaluating the capability of osteogenic cells to promote bone repair. Interestingly, a recent study has evaluated osteogenic potential of hiPSCs in comparison with BM-MSCs, which found that osteoinduced hiPSCs demonstrated comparable capability to repair rat calvarial defect and radial segmental bone defect as BM-MSC-derived osteoprogenitor cells, although *in vitro* results showed inferior osteogenesis of these differentiated hiPSCs compared to BM-MSC-derived osteoprogenitor cells^12^. Similarly, another study investigated osteogenic activity of several osteogenic differentiated hiPSCs *in vitro* and *in vivo*, which found that true bone formation of osteogenic cells derived from some hiPSC lines *in vivo* is not predicted by their poor osteogenic activity *in vitro*^11^. These studies suggest that osteogenic activity of implanted cells is not the only determinant for bone formation *in vivo*. Unfortunately, the angiogenic potential of these osteogenic differentiated cells was not assessed in these studies. Combined with these previous studies, our results emphasize the key connection between angiogenesis and osteogenic repair where implanted human cells recruit host vascular cells necessary to mediate effective *in vivo* repair.

While hESCs and hiPSCs have similar pluripotent potential, there are distinct differences between these cells, such as a unique gene expression signatures, different DNA methylation patterns, and metabolomic signatures^57^,^58^,^59^. In addition, genetic instability and chromosomal aberrations occur more frequently in low-passage of hiPSCs in contrast to low-passage of hESCs^13^. Transient memory of the transcriptional and epigenetic patterns in their cells of origin may also influence the differentiation of hiPSCs, but this influence is controversial^11^,^60^,^61^,^62^. Since continuous passaging of hiPSCs gradually eliminates the epigenetic memory effect, we used higher passages of these two hiPSCs in our study. Our results show similar pluripotency of hiPSCs with hESCs in terms of expression of pluripotent genes and typical surface markers. Since multiple factors may affect the differentiation potential of hiPSCs, such as the number of reprogramming factors, cell origin, or even the clones of hiPSCs^63^,^64^,^65^,^66^, our study compared the differentiation efficiency of two hiPSC lines reprogrammed from two different cells, peripheral blood mononuclear cells and umbilical cord blood CD34^+^ cells, using two different reprogramming approaches. Time course study of their differentiation profiles in optimized osteogenic conditions demonstrated similar differentiation efficiency in parallel with that of hESCs, although the differentiation efficiency of UCBiPSCs is a little bit lower in early passages (Fig. 1b). Homogenous populations of osteogenic cells are obtained from hESCs, UCBiPSCs and PBiPSCs after serial passaging, and phenotype of their osteogenic derivative cells are comparable with BM-MSC-derived osteoprogenitor cells. These results suggest that osteogenic differentiation of these two hiPSC lines are not influenced by either cell origin and reprogramming factors/approaches, which is consistent with other reports^11^,^62^. However, although the osteogenic differentiation efficiency and the phenotypes of their osteogenic derivatives are similar between these two hiPSC lines, the pro-angiogenic activities of their derivatives are quite different, leading to distinctly different capabilities to repair bone *in vivo*. Additional studies are required to better define why such a difference in angiogenic activity develops in cells produced from these different stem cell populations. Because of the variation between different clones of hiPSCs and between hiPSCs reprogrammed from various cell types, more clones of the hiPSC from the same cell type and more hiPSCs derived from different cell types are needed for future studies to better validate the developmental and regenerative differences between different hESC- and hiPSC-derived cells.

In conclusion, our study demonstrates that phenotypes of the osteoprogenitor cells derived from hESCs and two hiPSCs lines are similar to osteogenic cells derived from BM-MSCs. However, the pro-angiogenic activity of these osteogenic differentiated cells is markedly different, resulting in distinct capability to repair non-union fracture *in vivo*. These studies highlight the predominant role of pro-angiogenic potential of the lineage-committed cells to determine the tissue repair facilitated by cell-based therapies.

## Materials and Methods

### Cell Culture and Differentiation

Two hiPSC lines were generated in our lab for this study, UCBiPSCs derived from umbilical cord blood CD34^+^ cells^38^ and PBiPSCs from peripheral blood mononuclear cells using Sendai Virus by cytoTune-iPSC reprogram kit (ThermoFisher Scientific). The karyotypes of these hiPSCs were tested and found to be normal after reprogramming. The pluripotency of these hiPSCs were confirmed by flow cytometry, immunocytostaining and PCR analysis, with results comparable to the H9 hESC line^9^ (Supplemental Fig. 1). Undifferentiated hESCs, UCBiPSCs and PBiPSCs were maintained as previously described^9^. Briefly, these cells were co-cultured with irradiated mouse embryonic fibroblasts (MEFs) in regular undifferentiation culture medium which was replaced every day. Human BM-MSCs were isolated from fresh bone marrow (AllCells) according to the recommended protocol and expanded in MSC culture media, consisting of 10% defined fetal bovine serum (FBS, Hyclone), 1% Penicillin/Strep, 1% MEM-NEAA, 2 mM L-Glutamine in α-MEM (Invitrogen), which was replaced every 2 ~ 3 days. All these cells were incubated at 37 °C in 5% CO2 at 95% humidity.

To induce osteogenic differentiation, hESCs and hiPSCs after p30, were cultured according to the protocol described in our previous study^9^. Briefly, undifferentiated cells were dissociated and replated on pre-coated culture flask in MSC culture media with osteogenic supplements (OS, 50 ug/ml ascorbic acid, 10 mM β–glycerophosphate and 100 nM dexamethasone, all from Sigma). The differentiation media was replaced every 3 days and the differentiated cells were passaged regularly at 80 ~ 90% confluence. At the same time, BM-MSCs were cultured in MSC media with or without OS, as positive and negative control, respectively. The osteogenic differentiated cells were referred to as hESC-OS, UCBiPSC-OS, PBiPSC-OS and BM-MSC-OS in the following studies.

### Immnocytochemical Staining and Flow Cytometric Analysis

To demonstrate pluripotency markers in the undifferentiated cells, live cells were fixed and permeablized before incubated with primary antibodies, anti-Nanog (AF1997, R&D Systems), SOX2 (MAB2018, R&D), Oct4 (MAB4401, Millipore) and Tra-1-81 (560161, BD Biosciences) antibodies in 5% BSA overnight, followed by secondary antibodies conjugated with Alexa-Fluor-488 (A-11008, or A-11029, ThermoFisher Scientific). Slides were covered by anti-fade mounting medium with DAPI (H-1200, Vector Laboratories) and visualized under Zeiss Axioplan fluorescence microscope (Carl Zeiss, NY).

To detect the surface markers by flow cytometry, cells were prepared and stained as previously described^8^,^9^ before reading with the fluorescent-activated cell-sorting facility (FACSCalibur, BD) of phycoerythrin (PE)-conjugated SSEA4 (560128, BD Biosciences) and Tra-160 (560884, BD Biosciences), or allophycocyanin (APC) or PE-conjugated CD31 (555446, BD Biosciences), CD34 (555824, BD Biosciences), CD44 (559942, BD Biosciences), CD73 (550257 BD Biosciences), CD105 (17-1057, eBioscience) and CD146 (550315, BD Biosciences). Staining of intracellular protein osteocalcin (IC1419P, R&D) was performed according to the manufacturer’s recommended protocol. These flow cytometry data was analyzed with FlowJo software (Tree Star, Ashland, OR).

### PCR and qRT-PCR Analysis

Total RNA and cDNA were prepared as previously described^9^. PCR was performed using Promega GoTaq Master Mix (Fisher Scientific) to evaluate the expression of pluripotency genes in undifferentiated hESCs and hiPSCs, in comparison with those in BM-MSCs. qRT-PCR was performed using the SYBR Green PCR Master Mix (Qiagen) per reaction according to the recommended conditions. The level of the target genes were correlated to the standard concentrations and normalized by the levels of GAPDH as an endogenous reference. Then, the expression levels were further normalized by the expression levels in control cells. The primers of target genes and their annealing temperatures are listed in Table 1.

### Functional Assays to Evaluate Phenotypes of Differentiated Cells

The osteogenic differentiated cells were fixed for analysis of mineral deposition by Von Kossa staining and Alizarin Red S staining as previously described^9^. In addition, ALP assay kit (Abcam) was used to quantitatively evaluate osteogenic activity of these differentiated cells at p6 on day 3 after seeding, according to the vendor recommended protocol. ALP activity in 2 × 10^5^ cells was measured by Infinite 200 Pro plate reader and analyzed by Magellan Software affiliated to the plate reader (TECAN, Mannedorf, Switzerland).

To evaluate the osteogenic specificity of these differentiated cells, the osteogenic differentiated hESCs, hiPSCs and BM-MSCs (p5 ~ 7) were cultured in StemPro^®^ Adipogenic differentiation medium (ThermoFisher Scientific) for 3 weeks according to the vendor’s recommended protocol; undifferentiated BM-MSCs were cultured in adipogenic differentiation medium or MSC culture medium as positive and negative controls. Then, these differentiated cells were fixed and permeablized before being stained with AdipoRed reagent (Lonza) to show lipid droplet, which was visualized under Nikon TS100 inverted microscope.

### Tubulogenesis of HUVECs in the conditioned medium from osteogenic differentiated cells

In order to evaluate pro-angiogenic potential of these osteogenic differentiated cells from BM-MSCs, hESCs, UCBiPSCs and PBiPSCs, conditioned medium of these cells was collected and applied to stimulate tubulogenesis of human umbilical veil endothelial cells (HUVECs) *in vitro*. Briefly, the same amount of differentiated cells (p5 ~ 7) were re-plated and cultured overnight to ensure cell attachment to culture dish before switching the culture medium to serum free medium for an additional 48 hours; the conditioned media was collected and filtered to culture HUVECs on growth factor-reduced Matrigel (Corning). 4 hours later, the live HUVECs were stained with calcein AM (Life Technologies, NY) and tubulogenesis was visualized under Zeiss Axioplan fluorescence microscope (Carl Zeiss, NY). Total tube length and branching points were quantitatively analyzed by ImageJ software (NIH open resource).

### ELISA analysis of soluble angiogenic factors in the conditioned medium

To quantitatively evaluate angiogenic factors secreted by these osteogenic cells, angiogenic ELISA kit (Signosis) was used to detect soluble human angiogenic factors in the conditioned medium according to the vendor’s recommended protocol. Based on the protein concentration, conditioned media with the same amount of protein were loaded in the provided 96-well plate pre-coated with the antibodies of eight angiogenic factors. The concentrations of these angiogenic factors were finally measured by Infinite 200 Pro plate reader and analyzed by Magellan Software affiliated with the plate reader (TECAN, Mannedorf, Switzerland).

### Preparation of cell-seeded constructs and rat femoral fracture model

Following the protocol described in our previous study^9^, we prepared the cell-seeded constructs for the *in vivo* study. Briefly, one million cells, p6 ~ 7 as single cell suspension in fibrinogen, were seeded on gelatin sponge (0.2 × 0.5 × 1.5 cm) and then clotted with thrombin. The cell-seeded constructs were cultured in osteogenic medium overnight to ensure cell ingrowth to the sponge before implantation.

The protocol of animal study was approved by the IACUC at both the Minneapolis Medical Research Foundation and University of Minnesota, and animal surgery was performed in accordance to the approved guidelines. Male immunodeficient nude rats (Charles River), aged 8–12 weeks, were used in this study, with 12 rats in each group. The non-union fracture model was created according to the protocol introduced in previous studies^39^,^40^. Briefly, the midshaft of the right femur was exposed through a lateral incision after anesthesia in a sterile setting. A femoral fracture was created by making an oblique complete osteotomy at the lower one third of femur shaft with an oscillating mini-saw. The periosteum was cauterized 2 mm proximally and distally from the fracture site. An intramedullary Kirschner wire was inserted in a retrograde manner to immobilize the fracture. The cell-seeded (or no-cell control) gelatin sponge was wrapped circumferentially around the immobilized femur at the level of the fracture. Free cage activity was allowed after surgery, and rats were euthanized at 10-weeks.

### X-ray and microCT evaluation of bone healing

X-ray examinations were performed under anesthesia at 4-, 8-, and 10-weeks post-surgery. The fracture images were blindly reviewed and scored by two individuals according to the radiographic scoring system adapted from other studies^67^ (Table 2). To quantitatively evaluate bone formation at 10-weeks, microCT scanning of the healing femur was performed using Siemens Inveon microCT scanner (Siemens, Malvern, PA) at the Center for Magnetic Resonance Research at the University of Minnesota. To avoid the interference of the metal material, the intramedullary Kirschner wires were removed carefully before scanning. The harvested rat femurs were placed in polypropylene tubes filled with 70% ethanol, aligned orthogonally to the X-ray beam, and scanned at 80 kv and 500 uA with 720 steps for 360 degree rotation in a resolution of 35.84 μm and the pixel matrix 2368 × 3264. The images were acquired by Inveon Acquisition Workplace 1.5 and reconstructed with Inveon Research Workplace 4.2 associated with the scanner. In order to quantify the ratio of bone volume to total tissue volume (BV/TV) and trabecular bone thickness in the healing area, a standardized region of interest (ROI) in the reconstructed images was determined 2 mm above and below the fracture site with manually corrected contours to cross the gap in non-union fractures.

### Histology and Immunochemical staining of the healing tissues

To evaluate the histology of healing tissue, retrieved bone specimens were fixed and decalcified before embedded in paraffin and sectioned longitudinally, which were stained with hematoxylin and eosin (H&E) and alcian blue. The histological staining was visualized and scanned using Panoptiq digital slide imaging system (ViewsIQ, Richmond, Canada).

For immunohistochemical staining, the paraffin-embedded specimen was sectioned and processed as previously described^9^. Briefly, tissue slides were deparaffinized and rehydrated before antigen retrieval and blocking with goat serum. Then, the slides were incubated with primary antibodies (Ab), either mouse-anti-human osteocalcin Ab (ab13421, Abcam), mouse-anti-rat osteocalcin Ab (M186, Takara), rabbit-anti-rat collagen-II Ab (AB2036, Millipore) or mouse-anti-rat endothelial cells (ab9774, Abcam), before being incubated with secondary antibody conjugated with Alexa-Fluro-488 (A-11008, or A-11029, ThermoFisher Scientific). Finally, the tissue slides were covered by anti-fade mounting medium with DAPI (H-1200, Vector Laboratories) and visualized under Zeiss Axioplan fluorescence microscope (Carl Zeiss, NY).

### Biomechanical tests of the healing femur

Biomechanical torsional failure tests were conducted on a hydraulic materials testing load frame (MTS 858, MTS Systems, Eden Prairie, MN) as described in previous study^68^. To prevent influence of the pin on measured failure properties, it was carefully removed prior to testing, while using an external jig to maintain alignment. The femurs were aligned along the axis of the femoral pin and potted in Lipowitz's alloy. This ensures that the rotational axis is coaxial with the axis of the intramedullary pin. The femurs were loaded in rotation control at 0.5 deg/sec until specimen failure was observed, as indicated by a sharp decrease in torque. Axial displacement was held constant during the test. Torque and angular displacement were collected at a sampling rate of 25 Hz throughout the test. A previously validated MATLAB routine (MathWorks, Natick, MA) was utilized to derive the failure torque and torsional stiffness based upon the angle-torque data.

### Statistical analysis

Each experiment was repeated at least three times biologically for statistical analysis in this study, if not stated otherwise. The results are presented as mean ± standard error of mean (SEM). One-way ANOVA with Tukey post-hoc tests were used for statistical analysis and p value less than 0.05 was considered significant. For *in vivo* study, all rats were randomly designated to the control and treated groups and post-surgery evaluations of fracture healing were performed in a blinded manner.

## Additional Information

**How to cite this article**: Zou, L. *et al*. Angiogenic activity mediates bone repair from human pluripotent stem cell-derived osteogenic cells. *Sci. Rep*. **6**, 22868; doi: 10.1038/srep22868 (2016).

## Supplementary Material

Supplementary Information

## Figures and Tables

**Table 1:** Human oligonucleotide primers for qRT-PCR.

Gene Names	Sequences (5′→3″)	Product size (bp)	Ta (°C)
*Oct4*	F- CGTGAAGCTGGAGAAGGAGAAGCTGR- CAAGGGCCGCAGCTTACACATGTTC	247	66
*SOX2*	F- GCACATGAACGGCTGGAGCAACGR- TGCTGCGAGTAGGACATGCTGTAGG	207	66
*NANOG*	F- ACCTTGGCTGCCGTCTCTGGR- AGCAAAGCCTCCCAATCCCAAACA	151	64
*KLF4*	F- GCAGCCACCTGGCGAGTCTGR- CCGCCAGCGGTTATTCGGGG	130	65
*RUNX2*	F- CCAAATTTGCCTAACCAGAAR- GCTCGATTGCAATTGTCTCT	335	56
*OCN*	F- CCCAGGCGCTACCTGTATCAAR- CTGGAGAGGAGCAGAACTGG	209	61
*SPARC*	F- GCTCCACCTGGACTACATCGR- GGAGAGGTACCCGTCAATGG	325	59
*VEGFa*	F- CTACCTCCACCATGCCAAGTR- GCAGTAGCTGCGCTGATAGA	109	59
*bFGF*	F- GGCTATGAAGGAAGATGGAAGATTR- TGCCACATACCAACTGGTGTATTT	130	62
*GAPDH*	F- CCACTCCTCCACCTTTGACR- ACCCTGTTGCTGTAGCCA	102	60

**Table 2:** Scoring of fracture healing in X-ray images.

Callus Formation	Fracture Line
0—no callus	0—apparent gap between fracture ends
1—trace amount, no bridging	1—obvious line
2—apparent, incomplete bridging	2—almost invisible
3—massive, complete bridging	3—complete disappear
4—homogeneous bone structure	
