# Supplementary material for: Angiogenic activity mediates bone repair from human pluripotent stem cell-derived osteogenic cells
Source: Sci Rep. 2016 Mar 16;6:22868. doi: 10.1038/srep22868 (PMC4793227; doi:10.1038/srep22868)
Supplement: Supplementary Information [file srep22868-s1.pdf]

Angiogenic activity mediates bone repair from human pluripotent stem cell-derived osteogenic cells

Li Zou[1], Qingshan Chen[2], Zachary Quanbeck[2], Joan E. Bechtold[2], *Dan S. Kaufman[1]
[1] Department of Medicine, University of Minnesota, Minneapolis, MN 55455, USA
[2] Excelen Center for Bone & Joint Research and Education, Minneapolis, MN, 55415, USA

*Corresponding author:
Dr. Dan S. Kaufman
Division of Hematology, Oncology and Transplantation
420 Delaware Street SE, MMC 480
Minneapolis, MN 55455
Office: 612-624-0922
Email: kaufm020@umn.edu

<u>Supplemental Results:</u>

--- Supplemental Figure Legends:

Supplemental Figure 1. Pluripotency of undifferentiated hESCs and hiPSCs. (a) Flow cytometric analysis of pluripotency markers, SSEA4 and Tra-160, on hESCs, UCBiPSC and PBiPSCs, with BM-MSCs as control. **(b)** Flow cytometric analysis of hematoendothelial markers, CD31 and CD34, on hESCs, UCBiPSC and PBiPSCs. **(c)** Immunostaining of Nanog, SOX2, Oct4 and Tra-1-81 in the cells (scale bar: 200 μm). **(d)** RT-PCR results to show pluripotency marker genes in hESCs, UCBiPSCs and PBiPSCs, with BM-MSCs as control. Representative results out of 3 independent experiments were shown here.

Supplemental Figure 2. qRT-PCR analysis of Osteonectin (*SPARC*) in differentiated hESCs and hiPSCs at different passages. The gene expression level was normalized by undifferentiated cells for each cell line, n= 3 independent experiments.

Supplemental Figure 3. Cell morphology of osteogenic differentiated cells at p6. The bright field images showed that the differentiated cells, derived from BM-MSCs, hESCs, UCiPSCs or PBiPSCs, are quite similar in their morphology, spreading out to form flat cells and adhere to the substrate (scale bar: 100 μm).

Supplemental Figure 4. Phenotypic analysis of osteogeneic differentiated cells. (a) Flow cytometric analysis of surface markers on the differentiated BM-MSC-OS, hESC-OS, UCBiPSC-OS and PBiPSC-OS cells at passage 6, a representative result from 4 independent experiments. **(b)** AdipoRed staining of adipgenic droplet in BM-MSC, BM-MSC-OS, hESC-OS, UCiPSC-OS and PBiPSC-OS cultured in adipogenic medium for 3 weeks, representative images from 3 independent experiments (scale bar: 100 μm).

Supplemental Figure 5. Rat femur fracture repaired by implanted cells. (a) X-ray images of healing rat femurs repaired by different cells at 4-, 8- and 10-weeks, one representative image out of 12 for each group. **(b)** 3D reconstruction of microCT images from healing femurs repaired by different cells at 10-weeks.

Supplemental Figures:

Supplemental Figure-1

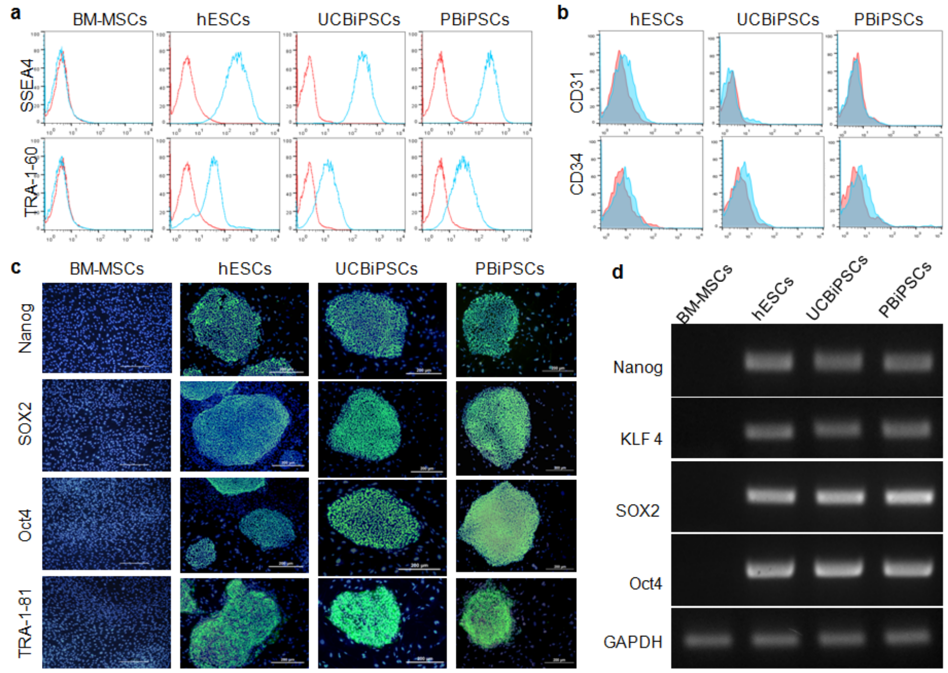

Supplemental Figure 1. Pluripotency of undifferentiated hESCs and hiPSCs. (a) Flow cytometric analysis of pluripotency markers, SSEA4 and Tra-160, on hESCs, UCBiPSC and PBiPSCs, with BM-MSCs as control. **(b)** Flow cytometric analysis of hematoendothelial markers, CD31 and CD34, on hESCs, UCBiPSC and PBiPSCs. **(c)** Immunostaining of Nanog, SOX2, Oct4 and Tra-1-81 in the cells (scale bar: 200 μm). **(d)** RT-PCR results to show pluripotency marker genes in hESCs, UCBiPSCs and PBiPSCs, with BM-MSCs as control. Representative results out of 3 independent experiments were shown here.

Supplemental Figure-2

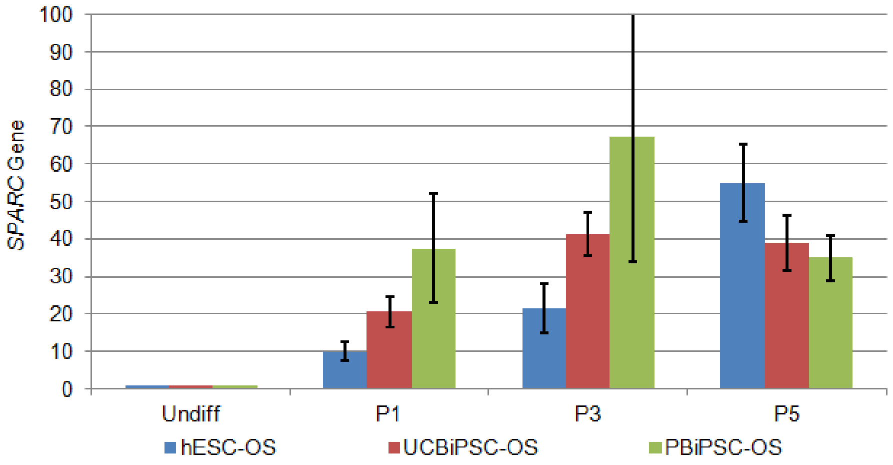

Supplemental Figure 2. qRT-PCR analysis of Osteonectin (*SPARC*) in differentiated hESCs and hiPSCs at different passages. The gene expression level was normalized by undifferentiated cells for each cell line, n= 3 independent experiments.

Supplemental Figure-3

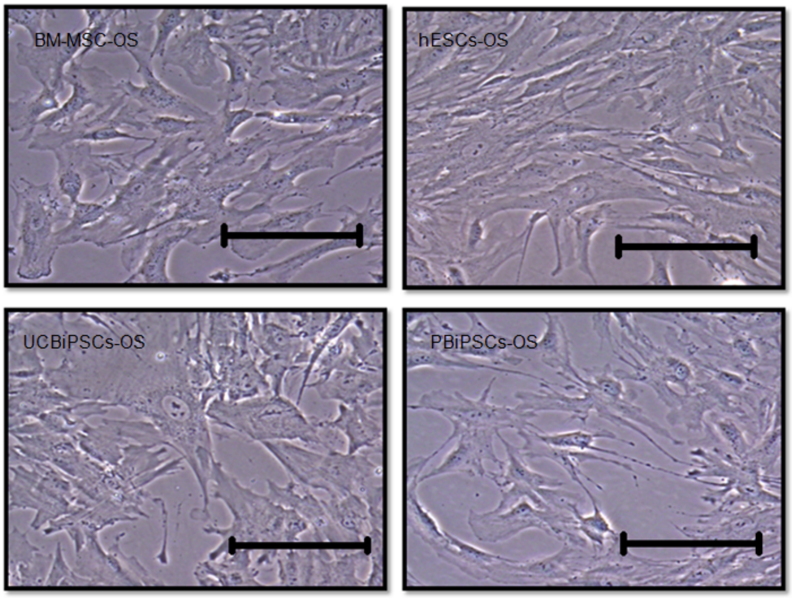

Supplemental Figure 3. Cell morphology of osteogenic differentiated cells at p6. The bright field images showed that the differentiated cells, derived from BM-MSCs, hESCs, UCiPSCs or PBiPSCs, are quite similar in their morphology, spreading out to form flat cells and adhere to the substrate (scale bar: 100 μm).

Supplemental Figure-4

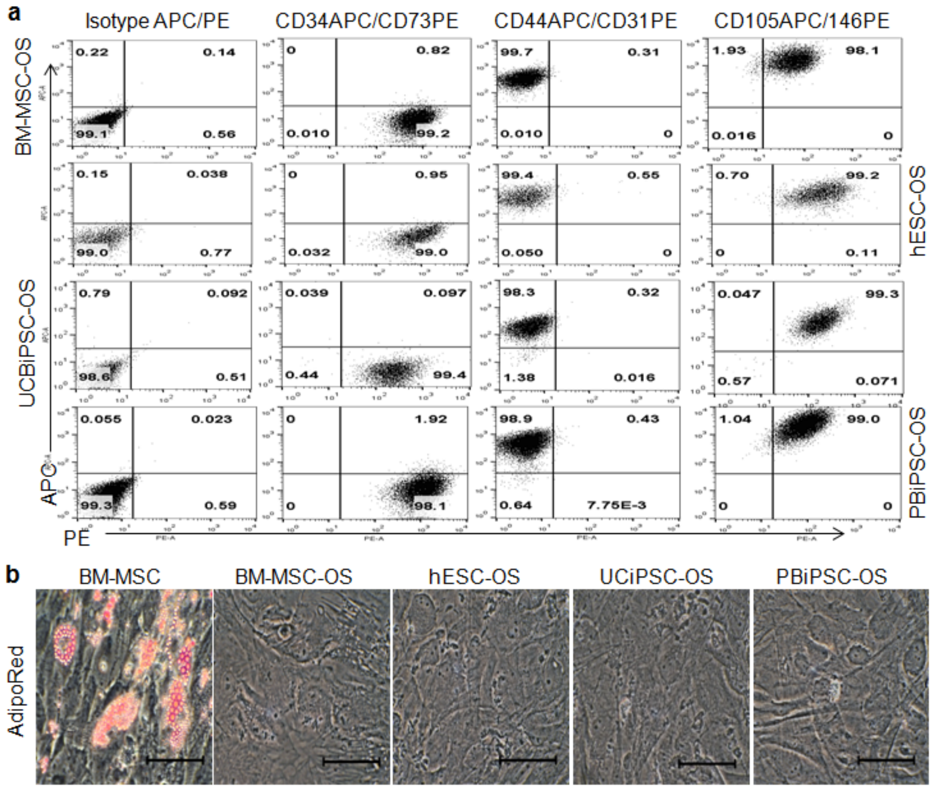

Supplemental Figure 4. Phenotypic analysis of osteogeneic differentiated cells. (a) Flow cytometric analysis of surface markers on the differentiated BM-MSC-OS, hESC-OS, UCBiPSC-OS and PBiPSC-OS cells at passage 6, a representative result from 4 independent experiments. **(b)** AdipoRed staining of adipgenic droplet in BM-MSC, BM-MSC-OS, hESC-OS, UCiPSC-OS and PBiPSC-OS cultured in adipogenic medium for 3 weeks, representative images from 3 independent experiments (scale bar: 100 μm).

Supplemental Figure-5

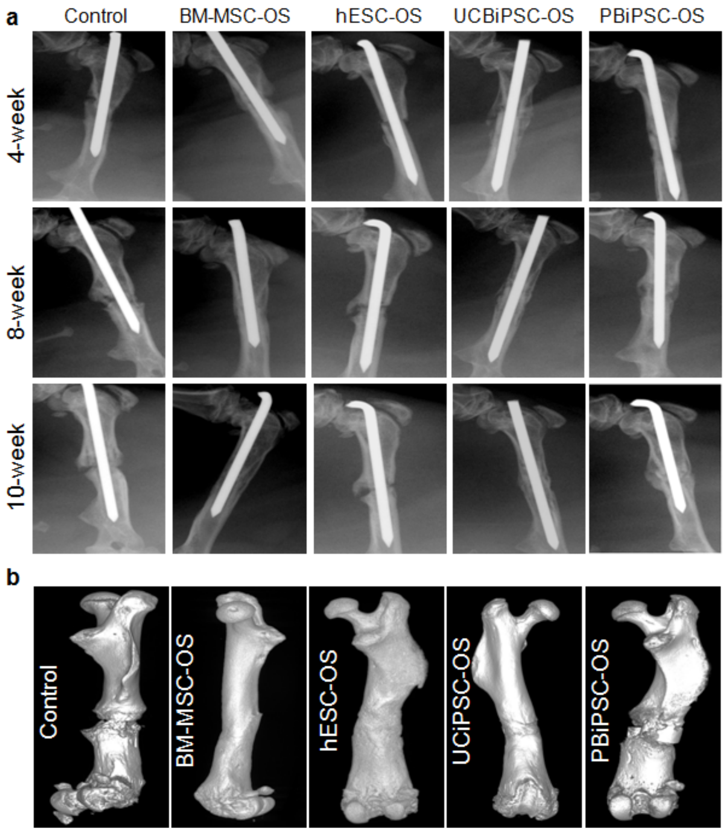

Supplemental Figure 5. Rat femur fracture repaired by implanted cells. (a) X-ray images of healing rat femurs repaired by different cells at 4-, 8- and 10-weeks, one representative image out of 12 for each group. **(b)** 3D reconstruction of microCT images from healing femurs repaired by different cells at 10-weeks.
